# The Extremely Rare Hypopharyngeal Fetal Rhabdomyoma in an Adult

**DOI:** 10.7759/cureus.18096

**Published:** 2021-09-19

**Authors:** Catarina Baraças, Mónica Farinha, Luis P Afonso, Maria T Bacelar

**Affiliations:** 1 Department of Radiology, Hospital Pedro Hispano, Unidade Local de Saúde de Matosinhos, Matosinhos, PRT; 2 Department of Pathology, Instituto Português de Oncologia do Porto, Oporto, PRT; 3 Department of Radiology, Instituto Português de Oncologia do Porto, Oporto, PRT

**Keywords:** head and neck tumor, pathological examination, head and neck imaging, hypopharynx, rhabdomyoma

## Abstract

Extracardiac rhabdomyomas are rare benign tumors showing skeletal muscle differentiation. They can be divided into adult, fetal, and genital subtypes.

Fetal rhabdomyomas are rarer than the adult subtype and although usually diagnosed at birth, the diagnosis is based on histology rather than patient age.

We present a rare case of a 25-year-old man with a cellular fetal (juvenile) rhabdomyoma, found in the postcricoid region of the hypopharynx.

## Introduction

Rhabdomyomas are rare benign mesenchymal lesions, showing cytologic features typical of striated muscle cells [1]. They are exceedingly rarer than rhabdomyosarcomas. Rhabdomyomas are divided into cardiac and extracardiac types, the former being more frequent.

Extracardiac rhabdomyomas can be of adult, fetal or genital subtype. Adult and fetal cases have a predilection for the head and neck region.

Fetal rhabdomyomas are extremely rare lesions usually discovered in the perinatal period and further divided into classic or myxoid and intermediate types. There are less than 30 cases reported in adult patients [2]. Cellular, juvenile, or intermediate types are terms used interchangeably and some authors advocate it as an intermediate form of striated muscle tumor between fetal and adult subtypes [3].

Malignant transformation of rhabdomyomas has not yet been described, although recurrences may occur if the lesion is not totally excised.

We present an extremely rare case of a young adult with a postcricoid cellular fetal rhabdomyoma, seen on computed tomography and magnetic resonance imaging. The lesion was excised and submitted to pathologic examination.

## Case presentation

A 25-year-old male presented with a six-month history of liquid and solid dysphagia. The patient also referred to dyspnea episodes that he attributed to upper respiratory tract infections. He had no relevant past medical or familial history.

Flexible laryngoscopy showed a lump in the postcricoid region of the hypopharynx, apparently reaching the right arytenoid cartilage and the right aryepiglottic fold, with bulging and decreased mobility of the ipsilateral hemilarynx.

**Figure 1:**
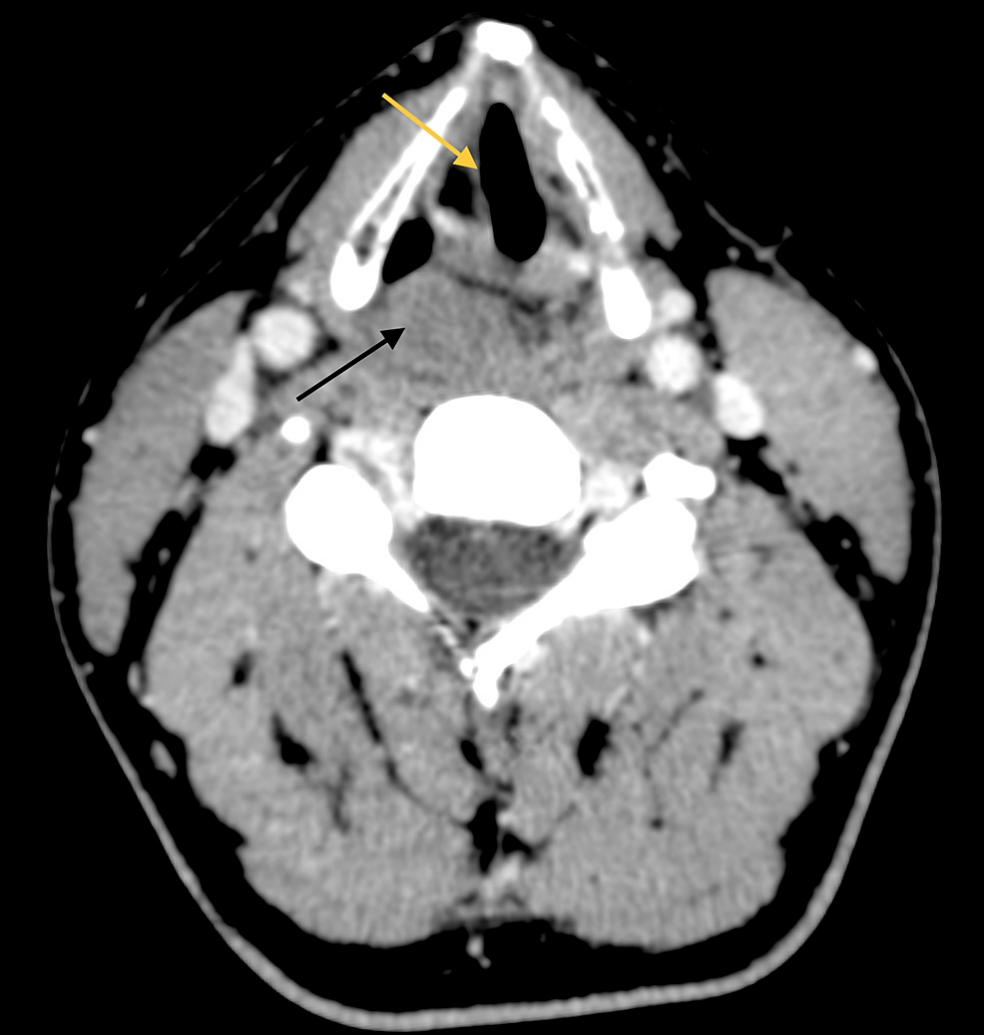
Axial contrast-enhanced computed tomography. Contrast-enhanced CT shows a well-circumscribed solid lesion in the right postcricoid region of the hypopharynx (black arrow). The lesion shows mild homogeneous enhancement. The right vocal cord is slightly medially deviated (yellow arrow).

A CT (Figure 1) showed a solid homogeneous submucosal lesion, with regular margins, and mild enhancement in the right postcricoid region of the hypopharynx. The right vocal cord was slightly medially deviated, although no cartilage erosion was seen.

**Figure 2:**
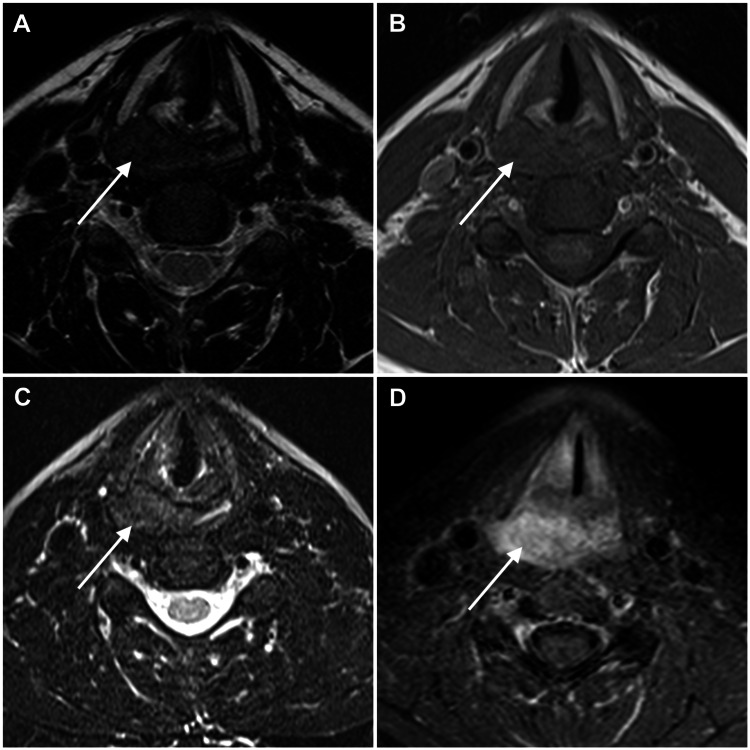
Magnetic resonance imaging of the lesion (white arrow). Both axial T2-weighted image (T2WI) (A) and fat-suppressed T2WI (C) showed a slightly hyperintense lesion to the muscle. On axial T1-weighted image (T1WI), the lesion (B) showed homogeneous isointense signal intensity compared to muscle. On axial T1 post-contrast image, the lesion had homogeneous enhancement.

An MRI (Figure 2) was performed confirming a submucosal, well-circumscribed, solid lesion in the postcricoid region of the hypopharynx. On T1-weighted images, the lesion showed homogeneous isointense signal intensity relative to muscle, and on T2-weighted images, the lesion was slightly hyperintense to the muscle. After gadolinium administration, the lesion enhanced homogeneously and progressively. No altered signal intensity was seen on the surrounding cartilages.

The patient was submitted to a partial pharyngectomy with a carbon dioxide laser.

Macroscopically, multiple reddish lesion fragments were observed and submitted in total for microscopic evaluation.

**Figure 3:**
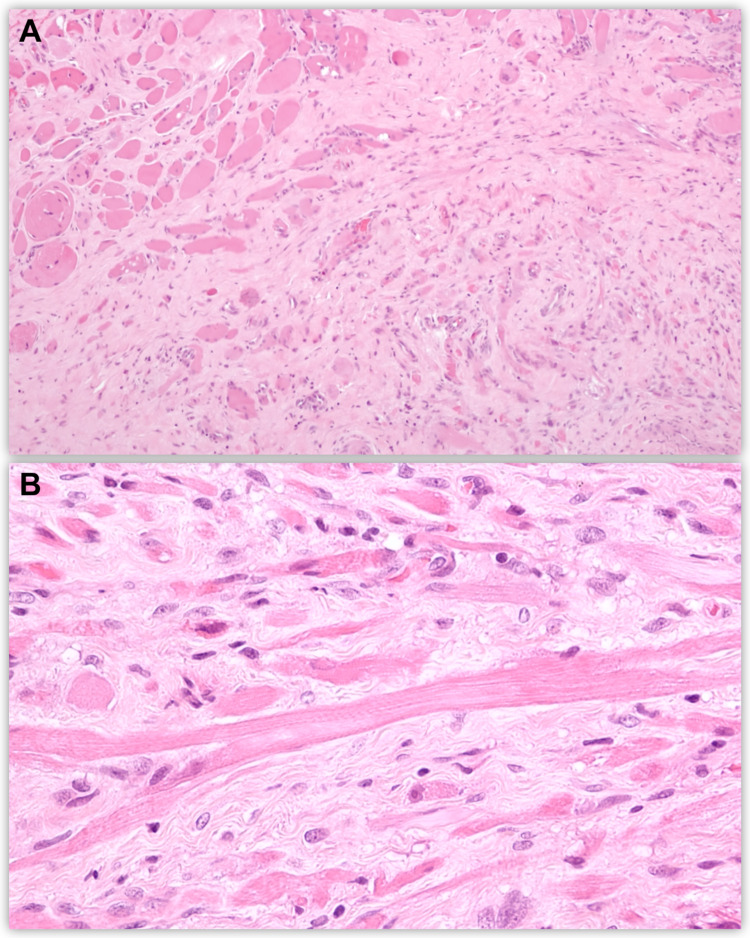
Histopathology findings of the lesion. (A) Irregular bundles of immature skeletal muscle fibers in a fibrous stroma focally infiltrating the normal adjacent skeletal muscle. (B) Strap-like rhabdomyoblasts blending with bland spindle cells and isolated cells with more of a rhabdoid appearance. There is mild atypia without mitosis.

Histopathology (Figure 3) revealed an unencapsulated lesion composed of bland spindle cells, immature elongated cells with bipolar cytoplasmic extensions, and strap-type rhabdomyoblasts with abundant eosinophilic cytoplasm and round vesicular nuclei displaying a fascicular growth pattern. Cross striations were easily identifiable. No pleomorphism, necrosis, or atypical mitoses were observed. The tumor cells showed strong cytoplasmic staining for desmin and multifocal myogenin and myogenic differentiation antigen 1 (MYOD1) expression.

The final diagnosis was an intermediate (juvenile) fetal rhabdomyoma.

After an initial six-month follow-up, a 12-month re-examination showed no relevant symptoms, although MRI exhibited what appears to be a persistence of the hypopharyngeal lesion. The multidisciplinary team decided to maintain a short-term follow-up.

## Discussion

Rhabdomyoma was first denominated by Zenker in 1864 [4], who intended to define a benign tumor composed of striated muscle tissue. Exceedingly rare, this entity represents only approximately 2% of all striated muscle tumors [5].

Cardiac rhabdomyomas are more frequent. An association with tuberous sclerosis is recognized [5]. Fetal rhabdomyoma occurs in association with nevoid basal cell carcinoma syndrome (Gorlin syndrome) where loss-of-function mutations in patched-1 (PTCH1) tumor suppressor gene are involved in the pathogenesis of these tumors.

Extracardiac rhabdomyomas can be divided into adult, fetal, and genital subtypes, according to the location and histological findings [6].

Adult subtype tends to occur after the age of 50, with a male predominance [7]. The majority of adult extracardiac rhabdomyomas occur in the head and neck area [7], predominantly in the pharynx and larynx. Solitary lesions are more frequent, but sometimes they can be multicentric [5].

Fetal rhabdomyoma can also be subdivided into classic and intermediate (or juvenile) forms. Intermediate rhabdomyomas represent a spectrum of differentiation and maturation between adult rhabdomyomas and classic fetal rhabdomyomas [1]. Fetal rhabdomyoma is even rarer than the adult subtype [8] and it is usually diagnosed at birth or even during the pregnancy, most frequently in the postauricular region. There are less than 30 cases reported in adult patients (Table 1) and none with the diagnosis of a fetal rhabdomyoma in the hypopharynx. It tends to present as a solitary, occasionally polypoid lesion with a male predominance.

**Table 1:** List of reported cases of fetal rhabdomyoma in adult patients.

Case number	Case reference	Age	Sex	Histologic type	Location
1	Misch [9]	21	F	Intermediate	Oral cavity (tongue)
2	Nath et al. [10]	38	F	N/A	Orbit
3	Dehner et al. [11]	56	M	Classic	Parotid
4	Sobel et al. [12]	39	F	N/A	Larynx
5	Ferlito et al. [13]	50	M	Classic	Larynx
6	Fu et al. [14]	60	M	Classic	Nasopharynx
7	Kapadia et al. [15]	54	M	Intermediate	Inferior limb (thigh)
8	Sant’agnese et al. [7]	53	M	Intermediate	Larynx (vocal cord)
9	Sant’agnese et al. [7]	37	M	Intermediate	Tongue
10	Sant’agnese et al. [7]	65	F	Classic	Larynx (vocal cord)
11	Sant’agnese et al. [7]	56	M	Intermediate	Neck
12	Kapadia et al. [15]	30	M	Intermediate	Eyebrow
13	Kapadia et al. [15]	20	F	Classic	Neck
14	Kapadia et al. [15]	58	M	Intermediate	Tongue
15	Kapadia et al. [15]	45	M	Intermediate	Face
16	Kapadia et al. [15]	20	F	Intermediate	Oral cavity (buccal mucosa)
17	Kapadia et al. [15]	37	M	Intermediate	Oropharynx (soft palate)
18	Kapadia et al. [15]	48	F	Intermediate	Larynx (vocal cord)
19	Hansen et al. [16]	55	M	N/A	Larynx
20	Wang et al. [17]	57	M	Classic	Oropharynx (tonsil)
21	Sharma et al. [18]	42	M	Intermediate	Larynx
22	González-Pérez et al. [19]	87	M	N/A	Bladder
23	Cai et al. [2]	37	F	Classic	Oropharynx (soft palate)
24 (present case)	N/A	25	M	Intermediate	Hypopharynx

Genital rhabdomyoma is a rare tumor typically found in females and is found in the vulva and vagina of middle-aged women.

Computed tomography imaging usually reveals a homogeneous lesion with mild to moderate enhancement, depending on the vascular stroma [20].

Magnetic resonance imaging shows a homogeneous isointense or slightly hyperintense lesion in muscles on T1 and T2-weighted images. The lesion enhances homogeneously.

Although the lesion has regular margins that are better seen after intravenous contrast administration, the isointensity to muscle signal can mimic malignant lesions.

Histologically, classic fetal rhabdomyomas are composed of bundles of immature skeletal muscle cells and myxoid stroma. The intermediate subtype of fetal rhabdomyomas shows greater cellularity with a less myxoid component, smooth muscle cells, and rhabdomyoblasts. These lesions lack nuclear atypia, infiltrative margins atypical mitoses, or necrosis. Immunohistochemistry shows strong positivity for desmin, with variable degrees of myogenin, MYOD1, and muscle-specific actin (MSA) expression.

The overall histological pattern and immunohistochemical profile of this case were diagnostic despite the older age of the patient.

Differential diagnoses of benign lesions in the hypopharynx include schwannomas, neurofibromas, granular cell tumors, paragangliomas, and vascular malformations, all of them are rare entities in adults [8]. Malignant lesions should always be ruled out, namely, rhabdomyosarcoma.

The most effective treatment implies the complete excision of the lesion. In the postcricoid region of the hypopharynx, it can be challenging, attending to the laryngeal apparatus proximity and to the potential loss of the organ’s function. Focal infiltration of skeletal muscle, as seen in this case, may contribute to the difficulty in achieving adequate margins.

Recurrences may develop in up to 40% of incompletely excised cases, particularly with adult rhabdomyomas [1]. Malignant transformation of rhabdomyomas has not yet been reported. 

## Conclusions

We report a case of an extremely rare lesion of cellular fetal rhabdomyoma, especially in adults. There are less than 30 cases reported in adult patients. To the best of our knowledge, we report the first case of a fetal rhabdomyoma of the hypopharynx diagnosed in adults. Imaging findings are unspecific and differential diagnosis is challenging. It is important to rule out characteristic malignancy features. Accurate histopathological assessment is necessary for correctly diagnosing this entity. Although a benign lesion, there can be recurrences, with complete excision being the most effective treatment.
